# Blood-Based Biomarkers Are Associated with Different Ischemic Stroke Mechanisms and Enable Rapid Classification between Cardioembolic and Atherosclerosis Etiologies

**DOI:** 10.3390/diagnostics10100804

**Published:** 2020-10-09

**Authors:** Dorin Harpaz, Raymond C. S. Seet, Robert S. Marks, Alfred I. Y. Tok

**Affiliations:** 1 School of Material Science & Engineering, Nanyang Technology University, 50 Nanyang Avenue, Singapore 639798, Singapore; miytok@ntu.edu.sg; 2 Department of Biotechnology Engineering, Ben-Gurion University of the Negev, Beer-Sheva 84105, Israel; rsmarks@bgu.ac.il; 3 Division of Neurology, Department of Medicine, Yong Loo Lin School of Medicine, National University of Singapore, NUHS Tower Block, 1E Kent Ridge Road, Singapore 119228, Singapore; mdcrscs@nus.edu.sg

**Keywords:** ischemic stroke mechanisms, diagnostics, etiology classification, blood-based biomarkers, statistical analysis, point-of-care biosensors

## Abstract

Stroke is a top leading cause of death, which occurs due to interference in the blood flow of the brain. Ischemic stroke (blockage) accounts for most cases (87%) and is further subtyped into cardioembolic, atherosclerosis, lacunar, other causes, and cryptogenic strokes. The main value of subtyping ischemic stroke patients is for a better therapeutic decision-making process. The current classification methods are complex and time-consuming (hours to days). Specific blood-based biomarker measurements have promising potential to improve ischemic stroke mechanism classification. Over the past decades, the hypothesis that different blood-based biomarkers are associated with different ischemic stroke mechanisms is increasingly investigated. This review presents the recent studies that investigated blood-based biomarker characteristics differentiation between ischemic stroke mechanisms. Different blood-based biomarkers are specifically discussed (b-type natriuretic peptide, d-dimer, c-reactive protein, tumor necrosis factor-α, interleukin-6, interleukin-1β, neutrophil–lymphocyte ratio, total cholesterol, triglycerides, low-density lipoprotein, high-density lipoprotein and apolipoprotein A), as well as the different cut-off values that may be useful in specific classifications for cardioembolic and atherosclerosis etiologies. Lastly, the structure of a point-of-care biosensor device is presented, as a measuring tool on-site. The information presented in this review will hopefully contribute to the major efforts to improve the care for stroke patients.

## 1. Introduction 

Stroke is a top leading cause of death, which occurs due to interference in the brain blood flow [1]. This interference is either due to ischemic stroke (blockage) in 87% of cases or due to hemorrhagic stroke (bleeding) in the remaining 13% of cases [2]. Ischemic stroke accounts for the majority of stroke cases, which makes ischemic stroke mechanism classification the second most important classification in stroke care [3]. There are four main ischemic stroke etiologies: 20% cardioembolic, 20% atherosclerosis (large artery disease), 25% lacunar (small vessel disease) and 5% other causes [4]. Additionally, 30% are termed cryptogenic strokes, which includes strokes from unknown causes [5,6]. The main value of subtyping ischemic stroke patients is for a better therapeutic decision-making process and to minimize time-to-thrombosis with the treatment of intervenors tissue plasminogen activator (IV-tPA) [4]. The average time is 3 h from stroke symptoms, and it was found useful only when administered within 4.5 h [7,8]. Anticoagulant therapy is the optimal treatment for cardioembolic stroke, while atherosclerosis stroke and other non-embolic etiologies are recommended to follow antiplatelet therapy [9]. A need to detect cardioembolic stroke patients still exists, which would benefit from anticoagulation treatment [10]. Brain cells die rapidly after stroke; therefore, treatment needs to be administered rapidly. “Time is brain” [11,12,13,14] because diagnostic time delays are associated with the worsened outcome of patients [15,16,17]. Stroke is more common in the elderly population (>65 years) [18,19,20,21], and has multiple reported risk factors including dyslipidaemia, hypertension, diabetes, obesity, dietary factors, inactive lifestyle, smoking and alcohol consumption [22,23]. Previous studies [24,25,26] have reported several differences between ischemic stroke etiologies. It was found that: (1) cardioembolic stroke is common in the elderly (>70 years), shows a smaller rate of secondary stroke, higher use of IV-tPA treatment, and the highest stroke severity; (2) atherosclerosis stroke is the most frequent stroke etiology in middle-aged patients (45–70 years), shows the highest rate of secondary stroke, the highest percentage of smoking, previous transient ischemic attack (TIA) and alcohol consumption; (3) lacunar stroke shows the highest rate of hypercholesterolemia, obesity, hypertension, diabetes mellitus and was associated with the lowest mortality and stroke severity. Since its development in 1993, the gold standard classification method for ischemic stroke etiologies is the Trial of Org 10172 in Acute Stroke Treatment (TOAST) classification [27,28,29,30]. It is mainly based on clinical symptoms and its reliability has been improved by the use of a computerized algorithm; however, the TOAST undetermined group is heterogonous because once a patient matches more than one possible etiology he is equally grouped as a patient with a no-cause identified or an incomplete investigation. Two additional classifications that are highly used including the National Institute of Neurological Disorders and Stroke (NINDS, Bethesda, MD, USA) classification [31] and The Oxford Community Stroke Project (OCSP) classification [32,33]. However, the current ischemic stroke mechanism classification methods are complex, time-consuming (between hours to days), and require professional personnel. 

Specific blood-based biomarker measurements have promising potential to improve ischemic stroke mechanisms classification [34,35,36]. Stroke is associated with several pathophysiological changes, which leads to the expression of different blood-based biomarker patterns [37,38,39,40,41,42,43,44,45,46,47,48,49]. A biomarker is a small molecule that is usually a protein, which can be easily detected in biofluids (e.g., blood, plasma, saliva, and urine) and provide vital information on the condition of a specific organ, disease, or treatment [50]. An ideal biomarker is specific, sensitive, and selective. Stroke biomarkers are from different identified origins: glial cells, neuronal cells, heart muscle cells (cardiomyocytes), blood vessels cells (myocytes), general inflammatory cytokines, cytoskeleton proteins, hemostatic proteins, lipids, metabolic proteins, and others [51]. The biochemical pathways are different among different ischemic stroke mechanisms (e.g., atherosclerosis versus cardiac embolism) [27,52,53,54,55,56]. Over the past decades, the hypothesis that different blood biomarkers are associated with different ischemic stroke mechanisms is increasingly investigated [35,57,58,59,60]. This review presents the recent studies that investigated blood-based biomarker characteristics differentiation between ischemic stroke mechanisms. The different blood-based biomarkers that best indicate different ischemic stroke etiologies are specifically discussed (Table 1), as well as the different cut-off values that may be useful in specific etiology classifications for cardioembolic (Table 2) and atherosclerosis (Table 3) etiologies. The following biomarkers are discussed: b-type natriuretic peptide (BNP), d-dimer, c-reactive protein (CRP), tumor necrosis factor-α (TNF-α), interleukin-6 (IL-6), interleukin-1β (IL-1β), neutrophil–lymphocyte ratio (NLR), total cholesterol, triglycerides, low-density lipoprotein (LDL), high-density lipoprotein (HDL) and apolipoprotein A. Lastly, the structure of a point-of-care biosensor device is presented, as a measuring tool for stroke biomarkers on-site. The information presented in this review will hopefully contribute to the major efforts that are being conducted to improve the care for stroke patients. 

## 2. Biomarkers that Best Indicate Cardioembolic Etiology

### 2.1. B-Type Natriuretic Peptide (BNP)

Over the past decades, b-type natriuretic peptide (BNP) and its derivative N-terminal fragment (NT-proBNP) have been reported as cardiovascular biomarkers, mainly for heart failure (HF) diagnosis as well as for stroke monitoring [125,126]. BNP (32 aa) is secreted by heart ventricles after excessive stretching of cardiomyocytes (heart muscle cells) [127]. NT-proBNP (76 aa) is secreted along with BNP and is biologically inactive [128]. BNP reflects on the ventricular condition with a half-life of 20 min, while NT-proBNP showed a half-life of 1–2 h [129]. Multiple studies reported that both BNP and NT-proBNP elevated serum levels are associated with cardioembolic stroke [61,62,63,64,65,66,67,68,69,70,71,72,73,74,75,76,77,78,79,80,81,82,83,84]. Additionally, they were found to be associated with atrial fibrillation (AF) condition, an abnormal heart rhythm (arrhythmia), that increases the risk of ischemic stroke and was also found to be associated with cardioembolic stroke [130]. Shibazaki K. et al. [71] reported that a BNP plasma level higher than 140 pg/mL is associated with patients with cardioembolic stroke, with a sensitivity = 80.5% and a specificity = 80.5%. Moreover, Kawase S. et al. [63] reported that the plasma level of BNP was higher in the case of cardioembolic stroke (366.6 pg/mL) compared to non-cardioembolic stroke (105.6 pg/mL; *p* < 0.01). Llombart V. et al. [64] reported that higher levels of BNP and NT-proBNP in the blood were significantly associated with cardioembolic stroke up to 72 h after stroke symptoms onset. Moreover, Chaudhuri J. R. et al. [61] reported that higher BNP levels were observed in 75% of cardioembolic stroke patients, while in the rest of the stroke etiologies: in 45.8% of lacunar patients, in 43.1% of atherosclerosis patients, and 34.5% of undetermined etiology. Moreover, Bai J. et al. [77] reported that both BNP (sensitivity = 65% (95% CI: 63%–68%) and specificity = 85% (95% CI: 83%–87%)) and NT-proBNP (sensitivity = 55% (95% CI: 52%–59%) and specificity = 93% (95% CI: 91%–94%)) were associated with cardioembolic stroke. Additionally, Nakamura M. et al. [79] reported that BNP predicted cardioembolic stroke (area under the receiver operating characteristic (AUC ROC) = 81%)). In addition to these findings, Wu Z. et al. [65] examined a point-of-care (POC) test for plasma BNP detection in the emergency department (ED), to promote initial recognition of cardioembolic stroke patients. BNP concentration was higher in the cardioembolic group (*p* < 0.01), a plasma BNP level higher than 66.5 pg/mL was associated with cardioembolic stroke (sensitivity = 76% and a specificity = 87%). In another study by Wu Z. et al. [74], the plasma BNP level was also measured at bedside. To conclude, BNP detection at bedside on admission can be added into stroke management as a strategy to improve the classification of stroke etiology.

### 2.2. D-Dimer

D-dimer is a by-product of fibrin degradation by plasmin and is a well-known hemostatic biomarker for blood coagulation activation and thrombosis, the formation of blood clot [131]. It was found useful for the diagnosis of venous thromboembolism and disseminated intravascular coagulation [132]. Multiple studies reported that elevated levels of D-dimer are associated with cardioembolic stroke [72,80,85,86,87,88,89,90,91,92,93,94,95,96,97,98]. Takano K. et al. [94] reported a D-dimer cut-off point of 300 ng/mL for distinguishing cardioembolic stroke from atherothrombotic infarction and lacunar infarction, with a sensitivity = 80% and a specificity = 77%. Additionally, Ageno W. et al. [85] reported that the optimal D-dimer cut-off point for discriminating between the presence and absence of a cardioembolic source is 2.00 mg/mL, with a specificity = 93%, a sensitivity = 59%, a positive predictive value = 73%, and a negative predictive value = 88%. Additionally, Dougu N. et al. [86] reported that D-dimer level higher than 1.6 mg/L (=1600 ng/mL) may indicate a high possibility of cardioembolic stroke. Moreover, Zi W. J. and J. Shuai [92] examined d-dimer levels measured upon admission (0–48 h from stroke symptom onset) and reported that median D-dimer levels were significantly (*p* = 0.000) higher in patients with cardioembolic stroke (2.17 mg/L (interquartile range (IQR), 1.24–3.48)), as compared with small-vessel occlusive stroke (0.59 mg/L (IQR, 0.25–0.96)), large-vessel occlusive stroke (0.56 mg/L (IQR, 0.22–0.95)), other stroke (0.25 mg/L (IQR, 0.14–0.41)) and unknown stroke (0.29 mg/L (IQR, 0.18–0.68)). D-dimer median level was higher in patients with cardioembolic strokes than in those with other etiologies (2.17 vs. 0.47 mg/L, *p* = 0.000). The receiver operating characteristic (ROC) analysis revealed the optimal cut-off value of plasma D-dimer levels as an indicator for diagnosis of cardioembolic strokes to be 0.91 mg/L (=910 ng/mL), with a sensitivity = 83.7% and a specificity = 81.5%, the area under the curve (AUC) = 86.2% (95% confidence interval (CI), 81.1%–91.2%). Additional studies reported different cut-off values of d-dimer level as an indication of cardioembolic stroke. Additionally, Liu L.-B. et al., [96] reported that D-dimer level higher than 791.30 ng/mL may indicate cardioembolic stroke, with a sensitivity = 58% and a specificity = 78%. Hirano K. et al. [93] reported that additional fibrin degradation products may also be associated with cardioembolic stroke. The levels of fibrin monomer complex (FMC), soluble fibrin (SF) and fibrin/fibrinogen degradation products (FDPs) were significantly higher in the cardioembolic group. The findings reported in these studies suggest that D-dimer levels in the acute stage of ischemic stroke might be useful in distinguishing cardioembolic stroke. Furthermore, the level of D-dimer is very rarely elevated in healthy individuals; however, it may increase in several illnesses and physiological conditions associated with thrombosis and thrombolysis [91].

### 2.3. Inflammatory Biomarkers: C-Reactive Protein (CRP), Tumor Necrosis Factor-α (TNF-α), Interleukin-6 (IL-6), Interleukin-1β (IL-1β)

Over the past years, evidence was found for the value of inflammatory biomarkers in cerebral cardioembolism, after inflammation was reported to be involved in atrial fibrillation [133] and thromboembolic events [134]. Several studies reported that elevated levels of the inflammatory biomarkers, c-reactive protein (CRP), tumor necrosis factor-α (TNF-α), interleukin-6 (IL-6), interleukin-1β (IL-1β), are associated with cardioembolic stroke [89,98,99,100,101,102,103,107,108,109]. Masotti L. et al. [99] examined CRP levels within 12 h from hospital admission among all ischemic stroke etiologies. The mean values of CRP were significantly higher in patients with cardioembolic stroke compared with an atherothrombotic large vessel and lacunar stroke. It was also previously reported that D-dimer itself stimulates monocyte synthesis and release of pro-inflammatory cytokines such as IL-6 [89]. Furthermore, Licata G. et al. [108] evaluated the plasma levels of immuno-inflammatory biomarkers in patients with cardioembolic ischemic stroke. Cardioembolic patients showed significantly higher median plasma levels of TNF-α (38.5 (22.2–46) pg/mL; *p* < 0.0001), IL-6 (11 (5.5–19) pg/mL; *p* = 0.0029) and IL1-β (11.5 (8–13) pg/mL; *p* < 0.0001). 

## 3. Biomarkers that Best Indicate Atherosclerosis Etiology 

### 3.1. Inflammatory Biomarkers: C-Reactive Protein (CRP) and Neutrophil–Lymphocyte Ratio (NLR)

Inflammatory biomarkers were previously found to be generally associated with atherosclerosis [135]. Higher CRP levels were found to be associated with atherosclerosis stroke [98,100,104,105,106]. Suwanwela N. C. et al. [100] examined the levels of inflammatory markers among patients with large vessel atherosclerosis (LAAS) and small vessel disease (lacunar stroke) and reported that higher CRP levels were significantly found in patients with large vessel disease. Additionally, Ladenvall C. et al. [104] reported that in the acute phase the CRP levels were higher in cardioembolic than in other etiologies (cardioembolic: 7.07 (2.39–17.8); atherosclerosis: 4.66 (1.79–13.9); lacunar: 3.08 (1.52–5.79)). While, after three months, the CRP levels were higher in atherosclerosis than in other etiologies (cardioembolic: 2.66 (1.07–5.46); atherosclerosis: 3.48 (1.42–9.99); lacunar: 2.65 (1.26–4.82)). Moreover, Zeng L. et al. [105] reported that the plasma levels of CRP were significantly (*p* < 0.05) higher in atherosclerosis etiology, with a cut off value of CRP ≥ 3.2 demonstrating 85.7% classification sensitivity. Moreover, neutrophil–lymphocyte ratio (NLR), which is calculated by dividing the concentration of neutrophils by the concentration of lymphocytes, was reported to indicate ischemic stroke from atherosclerosis etiology, as well as in cardioembolic etiology [110,111,112,113,114,115,116]. NLR is an inflammatory marker that has been recently introduced and examined in several coronary artery disease studies [136,137,138,139,140]. Elkind M. S. et al. [111] reported higher leukocyte counts in cardioembolic and atherosclerotic stroke etiologies. Moreover, Guven H. et al. [112] reported both higher leukocyte and neutrophil counts in great artery atherosclerosis, while the counts were lower in the lacunar group. Additionally, Gokhan S. et al. [110] reported that the NLR ratio level was significantly (*p* < 0.001) higher in the great artery atherosclerosis or atherothrombosis group (6.67 ± 3.74) compared to the other etiologies groups (cardioembolic: 1.74 ± 0.40; lacunar: 3.75 ± 1.74; unknown origin: 3.00 ± 1.49). Tokgoz S. et al. [113] also reported that the NLR was significantly higher (*p* = 0.001) in both the atherosclerotic (6.5 (IQR 7.2)) and cardioembolic (7.5 (IQR 8.9)) stroke subgroups than the lacunar infarct (3.20 (IQR 3.50)) subgroup. Lök U. and U. Gülaçti [115] reported that the NLR level did not vary significantly among the stroke subtypes (*p* = 0.070), while the neutrophil counts (10^3^/µL) were significantly (*p* = 0.008) different (large-artery disease (5.3 ± 1.5); cardioembolic (8.9 ± 4.01); lacunar (6.1 ± 2.0) and undetermined (7.1 ± 3.6)). Domaç, F. et al. [114] reported that the NLR levels in atherothrombotic and cardioembolic groups were 3.26 ± 2.35 and 4.46 ± 5.6, respectively, and this was found to be statistically significant (*p* = 0.03). 

### 3.2. Common Lipid Panel: Total Cholesterol, Triglycerides, Low-Density Lipoprotein (LDL), and High-Density Lipoprotein (HDL)

Serum lipids have been investigated for their association with different ischemic stroke mechanisms [117,118,119,120,121,122]. Iskra T. et al. [117] reported that LDL-B is also more prevalent in patients with atherosclerosis. Moreover, Slowik A. et al. [120] reported that patients with atherosclerosis had significantly higher concentrations of LDL than control (*p* < 0.05), and had higher concentrations of triglycerides and lower concentrations of HDL than patients with lacunar and control (*p* < 0.05). LDL phenotype B was more frequent in patients with atherosclerosis (63.3%) than in patients with lacunar (39.0%) or in control (16.7%) (*p* < 0.05). Additionally, Laloux P. et al. [119] investigated whether the lipid profile (total cholesterol, LDL, HDL, and triglycerides) of patients with atherosclerosis etiology is different than that of patients with lacunar etiology, which are both responsible for atherothrombotic cerebral ischemia. The study cohort (*n* = 485) was further selected into 240 patients with ischemic stroke (*n* = 182) or transient ischemic attack (*n* = 58) with a single classified etiology (61 patients with atherosclerosis, 65 with lacunar and 114 with cardioembolic). The levels of total cholesterol were significantly higher in patients with lacunar (*p* = 0.005) and atherosclerosis (*p* = 0.018) as compared to controls. The levels of triglycerides were significantly higher among all stroke etiologies as compared to controls (cardioembolic, *p* = 0.037; lacunar, *p* < 0.001; atherosclerosis, *p* = 0.014). Patients with atherosclerosis etiology showed a significantly higher LDL levels (*p* < 0.004) and lower HDL levels (*p* = 0.001). Only HDL levels were significantly (*p* = 0.047) different between patients with atherosclerosis etiology and with lacunar etiology, also in adjusted logistic regression analysis. Additionally, Bang O. Y. et al. [118] investigated the association of large artery atherosclerotic stroke with common serum lipid panel, including total cholesterol, triglycerides, low-density lipoprotein (LDL), high-density lipoprotein (HDL), and triglyceride. The study cohort included a large stroke patient number (*n* = 1049), classified to the following etiologies: 247 (23.5%) atherosclerosis, 224 (21.4%) lacunar, and 578 (55%) were non-atherosclerosis and non-lacunar group. The levels of total cholesterol, triglycerides, LDL, non-high-density lipoprotein cholesterol (HDL), and triglyceride:HDL ratio was significantly higher in atherosclerosis vs. non-atherosclerosis and non-lacunar patients, while the lipids levels were similar between atherosclerosis and lacunar patients. After adjustments, significant odds ratios (ORs) for atherosclerosis compared with all other ischemic stroke subtypes were triglycerides (OR 2.69, 95% CI 1.44 to 5.02) and non-HDL (OR 2.39, 95% CI 1.40 to 4.11). LDL was not found to be associated with atherosclerosis etiology. Moreover, Cui R. et al., [122] reported the hazard ratios of ischemic stroke with serum total cholesterol levels among large-artery patients for men (2.86 (1.31–6.27)) and women (0.75 (0.28–2.01)), while they were not associated with risk of lacunar or embolic infarction. Additionally, Yuan B.-B. et al. [121] reported that the levels of total cholesterol, triglycerides, LDL, apolipoprotein A, apolipoprotein B, apolipoprotein E, and lipoprotein A were higher and HDL was lower in stroke patients with atherosclerosis etiology as compared to the cardioembolic etiology. The levels of lipoprotein A, total cholesterol, and total cholesterol/HDL were higher in patients with cardioembolic etiology as compared to the lacunar etiology. The levels of total cholesterol, triglycerides, LDL, apolipoprotein A, apolipoprotein B, apolipoprotein E, lipoprotein A, total cholesterol/HDL, and LDL/HDL were all significantly different between patients with atherosclerosis etiology as compared to that with lacunar etiology. The levels of total cholesterol/HDL were different among all stroke etiologies, and the patients with atherosclerosis etiology showed the highest level. 

### 3.3. Apolipoprotein A

Apolipoprotein A belongs to the group of low-density lipoprotein (LDL), and it is associated with ischemic stroke from atherosclerosis etiology [117,123,124]. Iskra T. et al. [117] investigated the levels of lipoprotein A in ischemic stroke patients. Serum lipoprotein A is a lipoprotein consisting of apolipoprotein A and apolipoprotein B-100. It was found that median concentrations of lipoprotein A were similar in all studied groups (8.4 mg/dL in all stroke patients; 10.85 mg/dL in large vessel disease; 7.7 mg/dL in small vessel disease and 6.3 mg/dL in controls; *p* = non-significant). The percentage of subjects with increased lipoprotein A concentrations (i.e., > 30 mg/dL) was greater in large vessel disease (36.7%) than in small vessel disease (12.2%; *p* < 0.05) and in controls (10%; *p* < 0.05). Additionally, Zambrelli E. et al. [123] investigated the levels of apolipoprotein A in ischemic stroke patients (*n* = 94), as well as its association with different etiologies and stroke severity, as compared to healthy control (188). It was found that subjects carrying at least one small apolipoprotein A isoform were at increased risk of atherothrombotic stroke (OR 7.1, 95% CI 2.8 to 17.5, *p* = 0.00001) but not of lacunar infarction (OR 1.1, 95% CI 0.5 to 2.7, *p* = 0.78). Multivariate logistic regression analysis revealed that in the atherothrombotic stroke group, the presence of at least one small-sized apolipoprotein A phenotype was associated with a National Institutes of Health Stroke Scale (NIHSS) score > or = 6 (OR 13.6, 95% CI 1.6 to 111.9, *p* = 0.015). They concluded that small apolipoprotein A isoforms distinguish atherothrombotic stroke from lacunar infarction and are associated with the severity of atherothrombotic stroke. Furthermore, Kim B. S. et al. [124] reported that lipoprotein A levels of atherosclerosis stroke (*n* = 281, 34.6 mg/dL) were significantly (*p* < 0.001) higher as compared to other stroke mechanisms (cardioembolic: *n* = 204, 29.2 mg/dL, lacunar: *n* = 261, 24.2 mg/dL, other determined: *n* = 74, 24.1 mg/dL, undetermined: *n* = 192, 27 mg/dL).

## 4. Point-of-Care Biosensor as On-Site Test for Blood-Based Biomarker Detection

A point-of-care (POC) biosensor is a rapid on-site test that provides results within minutes. It is usually easy to operate, has a robust setup, and requires a small sample volume without any pretreatment [141,142]. Two famous examples of successful POC biosensors are the lateral-flow pregnancy test and the glucometer [143]. Routinely, biomarkers are usually detected by an enzyme-linked immunosorbent assay (ELISA), which is the most common immunoassay methodology [144]. It obtains highly sensitivity results; however, it is complicated, time-consuming, and consists of multiple steps. A POC biosensor may provide a more practical solution while still obtaining sufficient sensitivity [145,146]. A biosensor consists of a biorecognition entity (e.g., antibodies, antigens, DNA/RNA, whole cells, enzymes, or biomimetic molecules), which enables the detection of the target biological molecule. The interface is the main structure of the biosensor that allows its function, and it is usually based on adsorption, cross-linking, covalent binding, entrapment, self-assembled monolayers, and bulk modifications. Lastly, the transducer allows the signal measurement that is from optical, electrochemical, or acoustic origin [147,148]. There are different POC biosensor analytical formats such as paper-based [149,150,151], optical [152,153], electrochemical [154] and microfluidics [155]. Most of the POC biosensor platforms allow the detection of a single chosen biomarker. The development of a multi-biomarker detection device is still mostly research-based. Additionally, a quantitative biomarker detection is needed that allows the identification of a specific cut-off value relevant in the clinical setting, which will enable an improved decision-making process. To develop multiplex and quantitative POC biosensors for stroke biomarker measurement, there is a need to engineer novel platforms. Various POC biosensors have been clinically tested for their usefulness in stroke care [156]. These POC biosensors include different well-known biomarker detection: coagulation profile (international normalized ratio (INR), activated partial thromboplastin time (APTT)), blood-count (platelet, leukocyte, and erythrocyte count), and blood-chemistry (hemoglobin, glucose, c-glutamyltransferase, and p-amylase test) [15,157,158,159,160]. Unfortunately, these valuable tools are still not fully incorporated into the current clinical practice due to the time-consuming diagnostic procedures. For example, most of the guidelines for the management of stroke patients indicate that thrombolytic therapy should not be delayed on the account of these mentioned test results, with the only exceptions being bleeding abnormality, thrombocytopenia, and admission of anticoagulants [161]. Currently, half of stroke patients are misdiagnosed in the ED [162], while the clinical management scheme is still solely based on the assessment of symptoms and stroke scale tools [163]. There is a need for immediate and unbiased tools for the monitoring of stroke patients.

## 5. Conclusions

This review presents the recent studies that investigated blood-based biomarker characteristics differentiation between ischemic stroke mechanisms. Different blood-based biomarkers are specifically discussed, as well as the different cut-off values that may be useful in specific etiology classifications for cardioembolic and atherosclerosis etiologies. Various blood-based biomarkers were found to be significantly associated with ischemic stroke from cardioembolic etiology including BNP/NT-proBNP, d-dimer, CRP, TNF-α, IL-6, and IL-1β. Furthermore, various blood-based biomarkers were found to be significantly associated with ischemic stroke from atherosclerosis etiology including CRP, NLR, total cholesterol, triglycerides, LDL, HDL, and apolipoprotein A. Increased research efforts are still needed to identify useful biomarkers to differentiate ischemic stroke from lacunar etiology [164,165]. Lastly, the structure of a POC biosensor device is presented, as a measuring tool for stroke biomarkers on-site. A POC biosensor is a rapid on-site test that provides results within minutes. It is usually easy to operate, has a robust setup, and requires a small sample volume without any pretreatment. To develop multiplex and quantitative POC biosensors for stroke biomarker measurement there is a need to engineer novel platforms. The information presented in this review will hopefully contribute to the major efforts that are conducted to improve the care for stroke patients.

## Figures and Tables

**Table 1:** Blood-based biomarkers that best classify ischemic stroke etiologies, ↑ elevated vs. ↓ reduced.

Biomarker	Cardioembolic	Atherosclerosis	Lacunar	Ref.
B-Type Natriuretic Peptide (BNP)	↑	↓	↓	[61,62,63,64,65,66,67,68,69,70,71,72,73,74,75,76,77,78,79,80,81,82,83,84]
D-Dimer	↑	↓	↓	[72,80,85,86,87,88,89,90,91,92,93,94,95,96,97,98]
C-Reactive Protein (CRP)	↑	↑	↓	[98,99,100,101,102,103,104,105,106]
Inflammatory Cytokines: Tumor Necrosis Factor-α (TNF-α), Interleukin-6 (IL-6), Interleukin-1β (IL-1β)	↑	↓	↓	[89,107,108,109]
Neutrophil Lymphocyte Ratio (NLR)	↑	↑	↓	[110,111,112,113,114,115,116]
Common Lipid Panel: Total Cholesterol, Triglycerides, Low-Density Lipoprotein (LDL), High-Density Lipoprotein (HDL) and Apo-lipoprotein A	↓	↑	↓	[117,118,119,120,121,122,123,124]

**Table 2:** Blood-based biomarkers that best indicate cardioembolic etiology.

Biomarker	Finding	Study Ref.
BNP (Cardiac Biomarker)	cardioembolic ≥ 140 pg/mL (sensitivity = 80.5% and a specificity = 80.5%)	Shibazaki K. et al. 2009 [71]
	cardioembolic = 366.6 pg/mL, vs. non-cardioembolic = 105.6 pg/mL (*p* < 0.01)	Kawase S. et al. 2015 [63]
	Cardioembolic ≥ 66.5 pg/mL (sensitivity = 76% and a specificity = 87%)	Wu Z. et al. 2015 [65]
	BNP predicted cardioembolic stroke (AUC ROC = 81%)	Nakamura M. et al. 2018 [79]
	BNP (sensitivity = 65% (95% CI: 63%–68%) and specificity = 85% (95% CI: 83%–87%)) and NT-proBNP (sensitivity = 55% (95% CI: 52%–59%) and specificity = 93% (95% CI: 91%–94%))	Bai J. et al. 2018 [77]
D-Dimer (Hemostatic Biomarker)	D-dimer ≥ 300 ng/mL for distinguishing cardioembolic stroke	Takano K. et al. 1992 [94]
	D-dimer ≥ 2.00 mg/mL for discriminating between the presence of a cardioembolic source (specificity = 93%, a sensitivity = 59%, a positive predictive value = 73% and a negative predictive value = 88%)	Ageno W. et al. 2002 [85]
	D-dimer ≥ 1.6 mg/L may indicate cardioembolic stroke	Dougu N. et al. 2008 [86]
	D-dimer = 2.17 mg/L (IQR, 1.24–3.48), median levels were significantly (*p* = 0.000) higher in patients with cardioembolic stroke	Zi W.J. and J. Shuai 2014 [92]
	D-dimer ≥ 791.30 ng/mL may indicate cardioembolic stroke (sensitivity = 58% and a specificity = 78%)	Liu L.-B. et al. 2015 [96]
CRP (Inflammatory Biomarker)	CRP mean values were significantly higher in patients with cardioembolic stroke	Masotti L. et al. 2005 [99]
TNF- α, IL-6 and IL1-β (Inflammatory Cytokines)	Cardioembolic patients showed significantly higher median plasma levels of TNF-α (38.5 (22.2–46) pg/mL; *p* < 0.0001), IL-6 (11 (5.5–19) pg/mL; *p* = 0.0029) and IL-1β (11.5 (8–13) pg/mL; *p* < 0.0001)	Licata G. et al. 2009 [108]

Abbreviations: interquartile range (IQR); b-type natriuretic peptide (BNP); c-reactive protein (CRP); tumor necrosis factor-α (TNF-α), interleukin-6 (IL-6), interleukin-1β (IL-1β).

**Table 3:** Blood-based biomarkers that best indicate atherosclerosis etiology.

Biomarker	Finding	Study Ref.
CRP (Inflammatory Biomarker)	Higher CRP levels were significantly found in patients with atherosclerosis as compared to lacunar	Suwanwela N.C. et al. 2006 [100]
	In the acute phase the CRP levels were higher in cardioembolic than in other etiologies (cardioembolic: 7.07 (2.39–17.8); atherosclerosis: 4.66 (1.79–13.9); lacunar: 3.08 (1.52–5.79)). While, after 3 months, the CRP levels were higher in atherosclerosis than in other etiologies (cardioembolic: 2.66 (1.07–5.46); atherosclerosis: 3.48 (1.42–9.99); lacunar: 2.65 (1.26–4.82))	Ladenvall C. et al. 2006 [104]
	The plasma levels of CRP were significantly (*p* < 0.05) higher in atherosclerosis etiology, with a cut off value of CRP ≥ 3.2 demonstrating 85.7% classification sensitivity	Zeng L. et al. 2013 [105]
NLR (Inflammatory Biomarker)	Higher leukocyte counts in cardioembolic and atherosclerotic stroke etiologies	Elkind M.S. et al. 2005 [111]
	Both higher leukocyte and neutrophil counts were shown in great artery atherosclerosis, while the counts were lower in the lacunar group	Guven H. et al. 2010 [112]
	NLR ratio level was significantly (*p* < 0.001) higher in the great artery atherosclerosis or atherothrombosis group (6.67 ± 3.74) compared to the other etiologies groups (cardioembolic: 1.74 ± 0.40; lacunar: 3.75 ± 1.74; unknown origin: 3.00 ± 1.49)	Gokhan S. et al. 2013 [110]
	NLR was significantly higher (*p* = 0.001) in both the atherosclerotic (6.5 (IQR 7.2)) and cardioembolic (7.5 (IQR 8.9)) stroke subgroups than the lacunar infarct (3.20 (IQR 3.50)) subgroup	Tokgoz S. et al. 2013 [113]
	NLR level did not vary significantly among the stroke subtypes (*p* = 0.070), while the neutrophil counts (10^3^/UL) were significantly (*p* = 0.008) different (large-artery disease (5.3 ± 1.5); cardioembolic (8.9 ± 4.01); lacunar (6.1 ± 2.0) and undetermined (7.1 ± 3.6))	Lök U. and U. Gülaçti 2016 [115]
	NLR levels in atherothrombotic and cardioembolic groups were 3.26 ± 2.35 and 4.46 ± 5.6, respectively, and this was found to be statistically significant (*p* = 0.03)	Domaç, F. et al. 2019 [114]
Common Lipid Panel: Total Cholesterol, Triglycerides, LDL, and HDL	LDL-B is more prevalent in patients with atherosclerosis	Iskra T. et al. 2002 [117]
	Patients with atherosclerosis had significantly higher concentrations of LDL than control (*p* < 0.05), higher concentrations of triglycerides, and lower concentrations of HDL than patients with lacunar and control (*p* < 0.05). LDL phenotype B was more frequent in patients with atherosclerosis (63.3%) than in patients with lacunar (39.0%) or in control (16.7%) (*p* < 0.05)	Slowik A. et al. 2003 [120]
	The levels of total cholesterol were significantly higher in patients with lacunar (*p* = 0.005) and atherosclerosis (*p* = 0.018) as compared to controls. Patients with atherosclerosis etiology showed a significantly higher LDL levels (*p* < 0.004) and lower HDL levels (*p* = 0.001)	Laloux P. et al. 2004 [119]
	HDL ratio was significantly higher in atherosclerosis vs. non-atherosclerosis and non-lacunar patients. After adjustments, significant ORs for atherosclerosis compared with all other ischemic stroke subtypes were triglycerides (OR 2.69, 95% CI 1.44 to 5.02) and non-HDL (OR 2.39, 95% CI 1.40 to 4.11)	Bang O.Y. et al. 2008 [118]
	Hazard ratios of ischemic stroke with serum total cholesterol levels among large-artery patients for men (2.86 (1.31–6.27)) and women (0.75 (0.28–2.01)), while they were not associated with risk of lacunar or embolic infarction	Cui R. et al. 2012 [122]
	The levels of total cholesterol, triglycerides, LDL, apolipoprotein A, apolipoprotein B, apolipoprotein E, and lipoprotein A were higher and HDL was lower in stroke patients with atherosclerosis etiology as compared to the cardioembolic etiology. The levels of lipoprotein A, total cholesterol, and total cholesterol/HDL were higher in patients with cardioembolic etiology as compared to the lacunar etiology. The levels of total cholesterol, triglycerides, LDL, apolipoprotein A, apolipoprotein B, apolipoprotein E, lipoprotein A, total cholesterol/HDL, and LDL/HDL were all significantly different between patients with atherosclerosis etiology as compared to that with lacunar etiology	Yuan B.-B. et al. 2015 [121]
Apo-lipoprotein A	The percentage of subjects with increased lipoprotein A concentrations (i.e., >30 mg/dL) was greater in large vessel disease (36.7%) than in small vessel disease (12.2%; *p* < 0.05) and in controls (10%; *p* < 0.05)	Iskra T. et al. 2002 [117]
	Subjects carrying at least one small Apo-lipoprotein A isoform were at increased risk of atherothrombotic stroke (OR 7.1, 95% CI 2.8 to 17.5, *p* = 0.00001) but not of lacunar infarction (OR 1.1, 95% CI 0.5 to 2.7, *p* = 0.78)	Zambrelli E. et al. 2005 [123]
	Lipoprotein A levels of atherosclerosis stroke (*n* = 281, 34.6 mg/dL) were significantly (*p* < 0.001) higher as compared to other stroke mechanisms (cardioembolic: *n* = 204, 29.2 mg/dL, lacunar: *n* = 261, 24.2 mg/dL, other determined: *n* = 74, 24.1 mg/dL, undetermined: *n* = 192, 27 mg/dL)	Kim B.S. et al. 2010 [124]

Abbreviations: c-reactive protein (CRP); neutrophil–lymphocyte ratio (NLR); low-density lipoprotein (LDL); high-density lipoprotein (HDL).
